# Beliefs, attitudes and perceptions to sun-tanning behaviour in the Norwegian population: a cross-sectional study using the health belief model

**DOI:** 10.1186/s12889-019-6503-0

**Published:** 2019-02-18

**Authors:** Hanne Stavenes Støle, Lill Tove Norvang Nilsen, Pål Joranger

**Affiliations:** 1 Department of Nursing and Health Promotion, OsloMet – Oslo Metropolitan University, Postboks 4, St. Olavs plass, 0130 Oslo, Norway; 2 Department of Radiation Applications, Norwegian Radiation Protection Authority, Grini næringspark 13, 1361 Østerås, Norway

**Keywords:** Sun behaviour, Skin cancer prevention, Melanoma, Health belief model, Empowerment

## Abstract

**Background:**

Norway has one of the highest incidences of melanoma in the world. It has been suggested that the majority of all skin cancers could be prevented by changes related to sun-tanning behaviour. This study explores the sun-tanning behaviour of the Norwegian population using a modified Health Belief Model (HBM). Increased knowledge about beliefs, attitudes and sun-tanning behaviour can provide information which may be useful for future sun protection interventions.

**Methods:**

In 2017, 1004 members of the Norwegian population completed cross-sectional online surveys. People who seek the sun for tanning purposes was the eligibility criterion for this study, reducing the study population to 569. With the aid of the constructs from the HBM, predictive factors explaining sun-tanning behaviour were determined using multivariate linear regression adjusted for demographics (gender, age, education and income). Furthermore, the predictor variables, empowerment and benefits of tanning, were added to the model.

**Results:**

Five of the constructs in the modified HBM showed significant correlation with sun-tanning behaviour using bivariate analysis. The strongest correlation was perceived barriers of sun protection (0.42), with the next strongest being the benefits of tanning (0.30). The modified model explained 31% of the variation in sun-tanning behaviour using multivariate analysis. Significant predictors from the HBM to sun-tanning behaviour were perceived barriers to sun protection (Beta = 0.36, *p* < 0.001) and the severity of melanoma (Beta = − 0.20, *p* < 0.001). In addition, empowerment (Beta = 0.05, *p* = 0.05) and the benefits of tanning (Beta = 0.28, *p* < 0.001) proved to be variables with significant effect on sun-tanning behaviour. The demographic factors age, education and income were also associated with sun-tanning behaviour (*p* < 0.05).

**Conclusion:**

Based on the results of this study, several factors in the modified HBM had a significant impact on Norwegians’ sun-tanning behaviour. The results indicate that future sun protection interventions should focus on reducing barriers in relation to sun protection behaviour, as well as emphasizing the severity of adverse tanning behaviour and melanoma. Efforts to alter the perceptions of the beneficial factors of tanning behaviour can also be appropriate in health promotion campaigns and interventions. Finally, implementing empowerment strategies could have a positive effect on promoting healthy sun-tanning behaviour.

**Electronic supplementary material:**

The online version of this article (10.1186/s12889-019-6503-0) contains supplementary material, which is available to authorized users.

## Background

Melanoma is the most aggressive form of skin cancer and incidences continue to rise worldwide [1]. The Global Burden of Disease Study shows that Norway is among the top five countries in the world in terms of incidence, mortality, and healthy life years lost due to melanoma [2]. It is one of the fastest increasing cancers in Norway and represents a major public health challenge [3, 4].

Ultraviolet radiation (UV) is the main cause of all types of skin cancer [1, 5–7]. The Nordic climate offers limited exposure to natural sun. It has therefore been suggested that the increase in melanoma incidences in Norway may be explained by changes in sunbathing habits, increased trips to warmer countries, men using less sun protection than women, and increased promiscuity amongst elderly people [8]. It is important for health communication researchers and practitioners to consider health beliefs and behaviour that can encourage safer sun-tanning behaviour, increase the use of sun protection and instigate early detection of skin cancer. Research aimed at investigating and clarifying sun protection behaviour is therefore essential [9].

Many of the previous studies within the field, have focused on objective risk factors and sun burn in relation to sun-tanning behaviour [4, 10–12]. As UV is the main known cause of melanoma, the potential for prevention is high [3, 13], and research suggests that the majority of all skin cancers could be prevented by behavioural change related to sun-tanning behaviour [11, 14]. This study contributes to the research field by exploring sun-tanning behaviour utilizing an explanatory theory, the Health Belief Model (HBM), modified by adding empowerment and the benefits of tanning as separate factors.

The HBM is one of the most extensively used theories in health behaviour research, also related to melanoma prevention and interventions to promote behavioural change [15, 16]. The HBM’s four primary constructs; severity, susceptibility, benefits and barriers, can be used to predict whether or not and why individuals take action to prevent, detect or control illness conditions [15]. In our study, self-efficacy, perceived empowerment and the benefits of tanning as an activity in itself, were added as separate constructs to the model.

In health promotion, the Worlds Health Organization explains empowerment “*as a process through which people gain greater control over decisions and actions affecting their health”* [17]. Empowerment can be seen as an intervention or a strategy to help people change behaviour that cause poor health conditions [18]. Successful adoption of an empowerment model in health promotion can be used to achieve positive health outcomes as well as being more efficient in attaining the important outcomes in prevention and management of disease [19]. To the best of our current knowledge, empowerment has not previously been implemented into the HBM.

This study explores different components affecting the Norwegians’ sun-tanning behaviour by utilizing a modified HBM. The aim is to (1) determine the HBM’s explanatory power on sun-tanning behaviour in a Norwegian population-based sample and (2) explore the effects of individual perceived empowerment on sun-tanning behaviour in the HBM.

## Methods

### Study design and participants

This cross-sectional study was carried out by collecting data through online surveys in November and December 2017. Norstat, a market research company, was used to perform the data collection [20]. A total of 3393 survey invitations were sent out to a randomized sample from Norstat’s web panel. Respondents were recruited according to gender, age and geographic region in order to enhance the representativeness of the sample from the Norwegian population. A total number of 1004 completed the questionnaire, giving a response rate of 33%. People who did not reply or finish the survey were replaced with people from the same category, in order to reduce some selection bias.

**Fig. 1:**
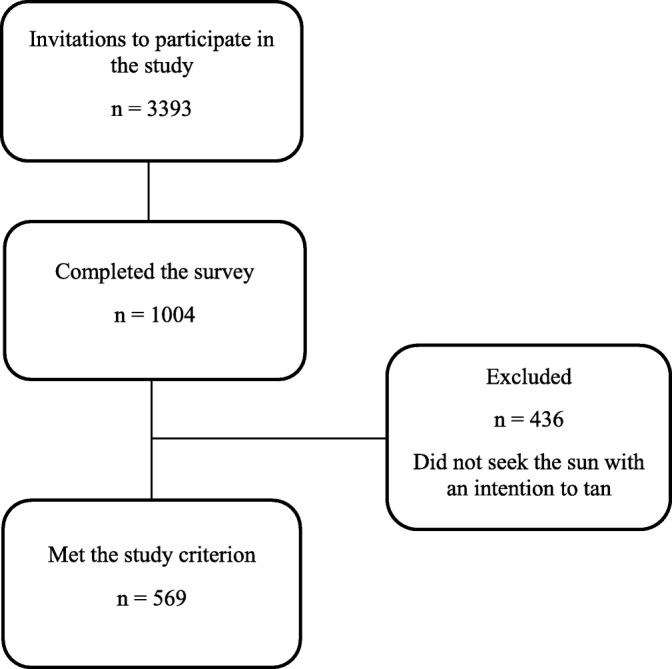
Flow diagram of the recruitment process

The eligibility criterion for the current study, was people who use the sun for tanning purposes. Of the 1004 respondents, 569 remained for data analysis (Fig. 1).

### Questionnaire

The self-reported questionnaire consisted of 43 questions in total and was tested in advance in order to determine how long time it would take. The results of the test showed that it took approximately 15 min to complete. Most questions were taken from previous studies [21–25] and some were translated from English or Danish to the Norwegian language. In addition, some new questions were especially developed for this particular study. The focus of the study was perceived and behavioural risk factors to melanoma and motivational/attitudinal factors to sun behaviour. The full questionnaire is available in Additional file 1: Appendix 1.

### The modified HBM

The HBM is a social-psychological model that attempts to explain and predict health behaviour by focusing on the individual’s attitudes and beliefs [26]. The model specifies that individuals are more likely to behave in a healthy manner if they first and foremost perceive that they are susceptible to a particular negative health outcome, especially if they perceive this outcome to be severe. The second reason as to why individuals behave healthily is if they perceive the benefits of healthy behaviour to be greater than the barriers related to the protective behaviour [16]. Finally, the individuals have to believe they can successfully perform the preventive action in order to reduce the threat.

**Fig. 2:**
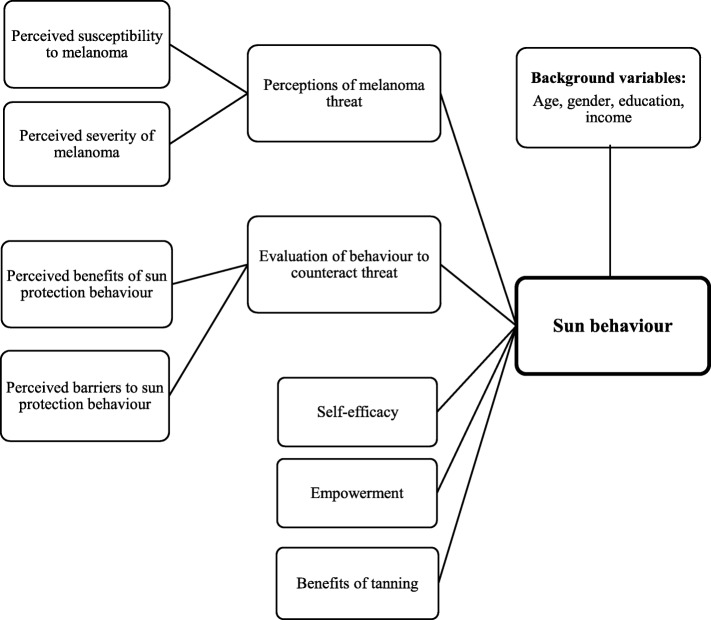
The modified Health Belief Model, showing all the predictor variables on sun-tanning behaviour

In this study, the questions were designed in order to test for five of the HBM constructs (Fig. 2). The first two constructs constitute the individual’s threat perception: perceived susceptibility and the severity of sunburn and subsequent melanoma. If these factors are high, the individual is likely to take health-related action [15]. The following constructs were perceived benefits and perceived barriers of sun protective-based behaviour. If the benefits outweigh the barriers, the behavioural change is more likely to occur [16]. The final HBM construct included was perceived self-efficacy, which focuses on the individual’s confidence of having a sun protective behaviour. In addition, we modified the HBM by including two extra variables, empowerment and perceived benefits of tanning as an activity in itself. All of the variables are set up as predictors to measure the individual’s sun-tanning behaviour.

### Variables and scales

Each prediction construct consisted of several questions, each having scaled answers in order to achieve a deeper and more complete overview. The outcome measure, sun-tanning behaviour, was constructed as an index of seven questions covering sun protection, sun exposure and tolerance of sunburn (Additional file 1: Appendix 1). Sun-tanning behaviour reflects perceived behaviour and not actual behaviour, as neither the exposure or use of protection are measured in this study. Some questions were measured on a five point Likert-type scale (never, rarely, sometimes, frequently, and always), and the other questions on a scale from 0 to 10, i.e., “totally disagree” to “totally agree”. Each of the seven questions in the dependent variable were given equal weight (0–1), so that the total score was in the range from 0 to 7.

The sum scale on sun-tanning behaviour ranged from low (low sun exposure, high protection behaviour and low tolerance of sunburn) to high (high sun exposure, low sun protection and high tolerance of sunburn). The higher the score, the greater the unhealthy sun related behaviour. In order to address the predictor variables of the HBM and the modified model, indexes on perceived susceptibility, severity, benefits, barriers, self-efficacy, empowerment and benefits of tanning were constructed (Table 1).

**Table 1:** The predictor variables in the modified HBM. Showing scale reliability, number of questions included in the indexes and examples of questions. The complete questionnaire with questions included in each predictor variable is available in Additional file 1

	Cronbach coefficient alpha (*α*)	Survey questions included	Examples
Susceptibility	0.62	3 items	*“How likely do you think it is that you will develop melanoma during your lifetime?”*
Severity	0.64	5 items	*“Getting melanoma in the future worries me” and “It is important for me to prevent getting melanoma”*
Benefits	0.76	6 items	*“Regular use of sunscreen with SPF 15 or more, is an effective way of avoiding sunburns”*
Barriers	0.81	14 items	*“When I am tanning, using sunscreen is uncomfortable”*
Self-efficacy	0.75	4 items	*“I am able to recognize warning signs of melanoma at an early stage”*
Empowerment	0.77	5 items	*“I am usually confident about the decisions I make” and “I feel powerless most of the time”*
Benefits of tanning	0.60	7 items	*“I sunbathe because I feel better with a tanned skin”*

### Validity and reliability

To minimize bias and improve the survey’s validity, fellow students and professional researchers tested the questionnaire throughout the development process. Feedback on wording, layout, questions and face validity improved the survey and contributed to the inclusion of important factors. A small pilot of the online survey was sent out to different age groups to check for overall understanding of the questions as well as to detect eventual deficiencies or misapprehensions in the survey.

The reliability of the predictor variables were tested by using Cronbach alpha (α). A value above 0.7 is commonly interpreted as an expression of good internal consistency [27]. Due to few questions in the indexes, we also used the corrected item-total correlation to assess reliability. This correlation should not include items with values below 0.3, which did not apply to any of the questions in this study.

### Statistical methods

Data were analysed in IBM Statistics SPSS version 24. Pearson’s correlations were performed to assess the associations between the modified HBM constructs and sun-tanning behaviour. A two-tailed *p*-value of *p* < 0.05 was considered statistically significant.

To test the prediction variables of sun-tanning behaviour, we used multivariate linear regression analysis. Regression coefficients (standard beta, Std. βs) and proportion of variance explained (R^2^) were calculated for several linear regression models. The predictor variables were entered in several steps. Preliminary analysis was conducted in order to ensure no violation of the assumptions of normality, linearity, multicollinearity and homoscedasticity. Furthermore, potential interaction between the predictor variables were examined and disproved.

## Results

### Study sample

One thousand four individuals completed the questionnaire. After excluding respondents who did not fulfill the eligibility criterion for intentional tanning, the final sample comprised 569 respondents (Fig. 1). The sample age range was 18 to 83 years, with a mean age of 48.6 (SD = 17.0). Age was recorded as a continuous variable (18 to 83 years old), then categorized into 18–29 years, 30–39 years, 40–49 years, 50–59 years, 60–69 years, 70+ years. The gender distribution changed from 50.8 to 58.3% percent females after excluding the respondents who did not meet the eligibility criterion. Furthermore, there were no large differences between the intentional tanning group and the total study sample regarding geographic distribution, educational level or income (Table 2).

**Table 2:** Selected characteristics for the study sample and total sample before exclusion criterion

	Study sample (*n* = 569)	Total sample (*n* = 1004)
	n (%)	n (%)
Gender		
Female	332 (58.3)	510 (50.8)
Male	237 (41.7)	494 (49.2)
Total	569 (100)	1004 (100)
Age		
18–29 years	98 (17.2)	164 (16.3)
30–39 years	89 (15.6)	149 (14.8)
40–49 years	110 (19.3)	177 (17.6)
50–59 years	92 (16.2)	149 (14.8)
60–69 years	107 (18.8)	204 (20.3)
70+ years	73 (12.8)	161 (16.0)
	*Mean: 48.6* *SD: 17.0* *Median: 49* *Min, max: 18, 83*	*Mean: 50.1* *SD: 17.5* *Median: 50* *Min, max: 18, 90*
Geographic region		
Northern Norway	53 (9.3)	98 (9.8)
Mid-Norway	89 (15.6)	150 (14.9)
Western Norway	121 (21.3)	215 (21.4)
Eastern Norway	187 (32.9)	336 (33.5)
Southern Norway including Telemark	45 (7.9)	83 (8.3)
Oslo	74 (13.0)	122 (12.2)
Educational level		
No education or less than 9-year elementary school		1 (0,1)
Primary school	17 (3.0)	36 (3.6)
High school	115 (20.2)	203 (20.2)
Diploma or vocational secondary education	74 (13.0)	138 (13.7)
University/College 1–4 years (Bachelor’s degree, cand.mag, or equivalent)	214 (37.6)	354 (35.3)
University/College 4 years or more (Master’s degree or equivalent)	119 (20,9)	217 (21.6)
University/College 6 years or more (PhD or equivalent)	18 (3.2)	35 (3.5)
Other	12 (2.1)	20 (2.0)
Income		
Under 200.000	77 (13.5)	138 (13.7)
200,000–299,999 NOK	46 (8.1)	111 (11.1)
300,000–399,999 NOK	99 (17.4)	175 (17.4)
400,000–499,999 NOK	137 (24.1)	216 (21.5)
500,000–749,999 NOK	153 (26.9)	264 (26.3)
750,000–999,999 NOK	35 (6.2)	61 (6.1)
1,000,000 NOK +	22 (3.9)	39 (3.9)

### Bivariate correlates of sun-tanning behaviour

The mean score on sun-tanning behaviour was 2.7 out of 7 (SD = 0.9). Regarding sun exposure, 42% of the respondents recalled tanning 2–3 days in the course of a week in Norway during the previous summer, 21% tanning for 4–5 days and 16% for 6–7 days a week. Furthermore, 44% of the respondents had been on a vacation in a sunny destination for two weeks or more within the past 12 months. The reported use of sunbeds was 11.5% between 1 and 9 times within the last 12 months and 4.8% 10–24 times. Sunscreen was the most preferred form for sun protection, with 73.5% of the respondents reported using sunscreen often or always. This percentage was 22.1% for clothes and 43.5% for shade.

In the HBM, perceived barriers of sun protection showed the strongest bivariate correlation with sun-tanning behaviour (0.42; Table 3). The subsequent strongest correlation was found between sun-tanning behaviour and perceived severity of melanoma. This showed a negative correlation (− 0.29) while the benefits of sun protection, also showed a weak negative correlation to sun behaviour (− 0.12).

**Table 3:** Bivariate analysis showing means and SD’s for sun behaviour, characteristics and HBM constructs and correlation between the variables (Pearson’s correlation)

	Range	Mean	SD	2	3	4	5	6	7	8
1. Sun behaviour	0–7	2.7	0.9	−0.29**	0.01	−0.12**	0.42**	−0.07*	−0.05	0.30**
2. Perceived severity	0–50	35.8	7.3		−0.04	0.27**	−0.36**	0.04	0.15**	0.11**
3. Perceived susceptibility	0–30	11.2	5.2			−0.05	0.10*	−0.24**	− 0.17**	0.00
4. Benefits of protection behaviour	0–24	18.2	3.3				−0.19**	0.24**	0.22**	0.17**
5. Barriers to protection behaviour	0–56	19.8	9.0					−0.10**	−0.28**	0.13**
6. Self-efficacy	0–40	20.9	7.8						0.31**	0.00
7. Empowerment	0–50	38.4	7.4							0.03
8. Benefits of tanning	0–24	16.6	2.9							

*Denotes a statistically significant two-tailed *p*-value < 0.05. **Denotes a statistically significant two-tailed *p*-value < 0.01

Empowerment and sun-tanning behaviour did not show a significant relationship (− 0.05). However, with the exception of the benefits of tanning, empowerment had a significant correlation with all the other predictor variables in the model. The benefits of tanning turned out to be one of the variables with the highest correlation to sun-tanning behaviour (0.30).

### Relationship between individuals’ characteristics and sun-tanning behaviour

Associations between HBM constructs and sample characteristics; gender, age, education and income, are displayed in Table 4. Total sun behaviour score among men was 2.9 out of 7 (SD = 0.1), which was higher than the female score of 2.6 (SD = 0.9). However, women use more sun protection measures that men (women 5.4 (SD = 2.2) compared to men 4.6 (SD = 2.0). The sum score of the questions on sun exposure (days in the sun in Norway, weeks of vacation abroad and use of sunbeds), revealed that men and women had the same score of 5.1 (SD = 2.3). Regarding the final question in the dependent variable, if it is worth getting sunburned to get a tan, women scored 2.9 (SD = 2.8) and men 3.5 (SD = 2.8).

**Table 4:** Relationship between sun behaviour and prediction variables according to gender, age, education and income with means, standard deviation (SD) and *p*-values based on Pearson’s correlation. Non-significant results are denoted N.s

Variables (Range)	Sun behaviour (0–7)	Perceived severity (0–50)	Perceived susceptibility (0–30)	Benefits of protection behaviour (0–24)	Barriers to protection behaviour (0–56)	Self-efficacy (0–40)	Empowerment (0–50)	Benefits of tanning (0–24)
	Mean (SD)	Mean (SD)	Mean (SD)	Mean (SD)	Mean (SD)	Mean (SD)	Mean (SD)	Mean (SD)
Gender	*p* < 0.01	*p* < 0.01	N.s	N.s	*p* < 0.01	N.s	N.s	*p* < 0.01
Female	2.6 (0.9)	37.2 (6.8)	11.4 (5.3)	18.3 (3.2)	18.4 (8.7)	37.0 (9.6)	38.4 (7.5)	16.9 (3.0)
Male	2.9 (1.0)	33.9 (7.6)	11.0 (5.2)	18.0 (3.5)	21.8 (9.0)	35.4 (9.5)	38.5 (7.4)	16.2 (2.7)
Age	N.s	p < 0.05	*p* < 0.01	N.s	*p* < 0.01	*p* < 0.01	p < 0.01	N.s
18–29 years	2.7 (1.0)	33.2 (7.6)	13.0 (5.3)	17.6 (3.4)	23.3 (9.3)	32.9 (9.4)	35.4 (8.0)	17.1 (3.1)
30–39 years	2.6 (0.9)	35.9 (7.4)	12.7 (4.7)	18.3 (3.1)	21.0 (8.2)	36.3 (8.2)	38.7 (7.4)	16.2 (3.2)
40–49 years	2.7 (1.0	36.4 (7.7)	12.8 (4.9)	17.6 (3.8)	20.5 (9.4)	35.7 (9.2)	38.6 (7.6)	16.4 (2.8)
50–59 years	2.8 (0.8)	36.7 (6.6)	10.3 (4.2)	19.1 (2.9)	17.9 (8.3)	38.4 (9.3)	39.4 (7.1)	17.0 (2.8)
60–69 years	2.9 (0.9)	37.0 (7.0)	9.8 (5.6)	18.6 (3.3)	17.2 (8.3)	37.6 (9.0)	39.5 (7.3)	16.5 (2.8)
70+ years	2.6 (1.0)	35.2 (7.1)	8.2 (5.0)	17.9 (3.1)	18.8 (9.1)	37.5 (11,8)	38.9 (6.3)	16.6 (2.5)
Educational level	p < 0.01	N.s	N.s	p < 0.05	N.s	p < 0.01	p < 0.01	p < 0.01
Primary school	2.8 (0,6)	36.9 (6.6)	10.5 (6.4)	17.5 (3.9)	19.1 (8.5)	34.7 (12.6)	35.2 (9.8)	17.1 (2.8)
High school	2.8 (1.0)	34.7 (7.5)	11.0 (5.5)	17.8 (3.5)	21.1 (9.1)	33.8 (10.4)	36.3 (7.9)	17.2 (3.1)
Diploma or vocational secondary education	2.9 (1.0)	35.5 (6.7)	10.7 (5.6)	17.5 (3.1)	20.7 (9.5)	35.2 (8.8)	38.1 (7.2)	16.7 (2.8)
University/College 1–4 years (bachelor’s degree, cand.mag, or equivalent)	2.7 (0.9)	35.9 (7.6)	11.8 (5.1)	18.5 (3.3)	19.4 (9.0)	37.5 (9.1)	39.2 (7.3)	16.6 (2.9)
University/College 4 years or more (Master’s degree or equivalent)	2.6 (0.9)	36.26 (7.3)	11.0 (5.0)	18.5 (3.1)	18.5 (8.5)	36.8 (9.6)	39.6 (6.6)	16.2 (2.6)
University/College 6 years or more (PhD or equivalent)	2.5 (1.2)	35.11 (6.7)	11.8 (4.4)	18.7 (3,5)	20.4 (6.4)	41.3 (7.4)	41.6 (4.7)	14.9 (2.8)
Other								
Income	N.s	N.s	N.s	N.s	p < 0.05	p < 0.01	p < 0.01	p < 0.05
Under 200.000	2.7 (1.1)	33.9 (7.6)	12.8 (5.2)	17.2 (4.0)	22.6 (9.1)	33.1 (8.6)	34.6 (8.0)	16.8 (3.2)
200,000–299,999 NOK	2.6 (1.0)	36.4 (7.4)	10.9 (5.3)	18.8 (3.3)	21.9 (10.1)	36.9 (11.4)	37.2 (9.0)	16.8 (2.6)
300,000–399,999 NOK	2.7 (0.9)	37.1 (7.0)	9.5 (5.3)	18.2 (2.8)	18.4 (9.4)	35.9 (9.6)	38.3 (7.5)	17.3 (2.9)
400,000–499,999 NOK	2.7 (0.9)	36.3 (7.1)	11.4 (5.2)	18.7 (3.1)	19.0 (8.4)	37.6 (9.9)	38.6 (6.8)	16.4 (3.1)
500,000–749,999 NOK	2.8 (0.9)	35.3 (7.6)	11.4 (5.2)	18.1 (3.4)	19.7 (8.7)	36.3 (9.2)	39.5 (6.7)	16.4 (2.7)
750,000–999,999 NOK	2.7 (0.8)	35.2 (6.8)	11.6 (5.0)	17.8 (3.5)	18.7 (9.3)	36.9 (8.4)	41.7 (5.8)	15.9 (2.5)
1,000,000 NOK +	2.9 (0.8)	35.9 (7.2)	11.8 (3.9)	18.3 (3.4)	19.8 (7.2)	39.6 (9.1)	40.8 (7.9)	16.6 (2.9)

Overall, Table 4 shows small variations within the different groups (gender, age, education and income), which indicate that the sample are generally homogenous. Though, some differences were found statistically significant. Perceived susceptibility to melanoma showed a decrease with age, where young people seemed to have a greater perceived susceptibility than the older respondents (*p* < 0.01). Perceived barriers to sun protection also decreased with age (*p* < 0.01). Respondents with higher education reported a lower sun behaviour score than people with lower education (p < 0.01). The benefits of tanning showed a decline as education increased (p < 0.01), as opposed to a slight increase in reported benefits of sun protection (*p* < 0.05).

Both the variables perceived self-efficacy and empowerment increased with level of education, income and age (*p* < 0.01).

### Test of the explanation effect of sun behaviour in the HBM

Multiple linear regression analyses were conducted in several steps in order to test the predictions on sun behaviour (Table 5). The first model only included the four main predictors of the HBM and showed a R^2^ of 19.9%. This model illustrated the amount of variance in the outcome variable, sun behaviour, that may be accounted for by the main predictor variables. When self-efficacy and descriptive variables of significance (gender, age, education and income) were included in the model, R^2^ increased to 20.1 and 23.2%, respectively. Finally, the model was modified by first implementing empowerment and thereafter the benefits of tanning. The full model, with all variables included, explained 31.1% of the variance in sun behaviour.

**Table 5:** Multivariate regression analysis testing associations between different predictor- and background variables in the HBM and sun behaviour with standard beta-values (Std. βs) and *p*-values

Variables	Model 1		Model 2		Model 3		Model 4		Model 5	
	Std. βs	*p*-value	Std. βs	*p*-value	Std. βs	*p*-value	Std. βs	*p*-value	Std. βs	*p*-value
*Constant (Unstandardized β)*	2.813	.000	2.898	.000	2.745	.000	2.388	.000	1.336	.000
Severity	−0.163	.000	−.165	.000	−.166	.000	−.170	.000	−.203	.000
Susceptibility	−0.031	.411	−.040	.303	.000	.992	.010	.812	.008	.833
Benefits of behaviour	−0.008	.841	.002	.970	.010	.804	−.001	.987	−.051	.193
Barriers to behaviour	0.361	.000	.359	.000	.380	.000	.400	.000	.335	.000
Self-Efficacy			−.040	.319	−.045	.261	−.067	.105	−.060	.127
Gender					.000	.997	−.001	.974	.029	.445
Age					.119	.005	.121	.004	.117	.004
Education					−.124	.004	−.131	.002	−.089	.032
Income					.102	.023	.087	.055	.086	.046
Empowerment							.099	.019	.078	.053
Benefits of tanning									.281	.000
R^2^	19.9%		20.1%		23.2%		24.0%		31.1%	

In model 5, the variables barriers, severity and the benefits of tanning showed a statistically significant impact on sun-tanning behaviour (*p* < 0.001). Perceived barriers to sun protection was the strongest predictor with β = 0.335. Barriers and severity were seen to be of high credibility with a significant beta value in all of the tested models (p < 0.001) and beta values ranging from 0.335 to 0.400 and − 0.163 to − 0.203, respectively. Empowerment showed marginally significant explanation of sun behaviour (β = 0.08, *p* = 0.053), while the benefits of tanning proved to be the variable with second strongest impact on sun behaviour (β = 0.281 and *p* < 0.001).

The background variables age, education and income showed a significant association to the model (*p* < 0.05). Geographic region was not included in any of the models, as no significant association was found between this variable and sun behaviour.

## Discussion

In this cross sectional-study the modified HBM’s explanatory power on sun-tanning behaviour in a Norwegian population-based sample was examined. The model with the four original constructs explained 19.9% of the variance in sun-tanning behaviour, whereas the full model which included self-efficacy, empowerment and the benefits of tanning, provided an explanation for 31.1% of the variance. The most significant primary predictor was perceived barriers to sun protection, followed by the benefits of sun-tanning and perceived severity of sunburn and melanoma. Empowerment was a significant prediction variable, indicating that an individuals’ perceived empowerment can have an effect on ones’ sun-tanning behaviour.

In contrast to other studies which have focused on sun protection behaviour and effects of UV-radiation [4, 11, 28, 29], our study examined the individual’s perceptions of attitudes and behaviour in relation to tanning utilizing a modified HBM. When predicting human behaviour using models, R^2^ values resulting in less than 50% is common [30], as also seen in our study with R^2^ just over 31%. The most important predictor, perceived barriers, showed consistently the greatest effect on sun-tanning behaviour in all of the tested models (Table 5). Furthermore, both barriers and severity were significant in all of the analysis and remained significant even when other factors were added to and affected the model. This illustrates the important impact these variables can have on sun-tanning behaviour.

Perceived barriers to sun protection behaviour was the predictor variable in our study with the most plausible clarifying effect on sun behaviour in both the bivariate and the multivariate analysis. This indicates that individuals who consider sun protection to be disadvantageous, may not use adequate sun protection although they have a high sun exposure behaviour. Our findings are in accordance with a meta-analysis that determined perceived barriers to be the strongest of the HBM dimensions across the various study designs and behaviour [16]. Other research also found barriers to sun protection to have one of the strongest impacts on individuals sun protective behaviour [10]. It has been suggested that individuals may fail to adopt healthy sun-tanning behaviour due to the expenses involved or as a result of the inconvenience of using sunscreen or clothes when in the sun [31–33].

Another important reason as to why barriers to sun protection are so high, is the beneficial effects of obtaining a sun tan. About 80% of the respondents in this study reported to feel and look better with a tan (data not shown). Another Norwegian survey reported that the intention of sunbathing, for both sexes, was to achieve a tan [34]. For many people, a sun tan is associated with physical and emotional health as well as attractiveness, and the benefit of tanned skin can be a motivating factor for intentional tanning [31]. Benefits of tanning turned out to be one of the variables in this study with the highest correlation to sun behaviour in both the bivariate and the multivariate analysis. This is both in accordance with and contradictory to other studies. One study claimed that the advantages of tanning predicted sun-tanning behaviour through intention to sunbathe, but also through intention to use sun protection [35]. Other studies also ascertained that the benefits of sun-tanning were one of the strongest predictors for sun protective behaviour [31, 36, 37]. Use of sun protection causes lower score on our sun-tanning behaviour score. Previous research has shown that individuals’ negative perception of the consequences of initial tanning were outweighed by the positive consequences [31], and likewise, immediate tanning was more appreciated than possible long-term consequences [31]. However, our findings also show a negative correlation between perceived severity and sun-tanning behaviour. This indicates that individuals who are worried about getting melanoma, and find it to be a serious disease, have healthier sun-tanning behaviour.

Contrary to previous findings, our study found a weak relation between susceptibility and sun-tanning behaviour. In a meta-analysis of the HBM susceptibility was not seen to be related to sun-tanning behaviour in the majority of studies [16]. However, other studies have found that perceived susceptibility to skin cancer was a powerful predictor of both intention to sun protect and to sunbathe [35, 38, 39]. In our study, neither susceptibility nor the benefits of protective behaviour had a significant effect on variance of sun behaviour in any of the models. Self-efficacy can be related to the individual’s ability to change behaviour. Previous studies have proposed that an individual’s level of self-efficacy and self-awareness are important constructs in terms of sun protective behaviour and beliefs [35, 40]. In this study self-efficacy showed a significant correlation to sun-tanning behaviour in the bivariate analysis but no significant effect in the multivariate analysis.

Due to the fact that empowerment is an important health-promoting factor, our study sought to explore the hypothesis that individuals with high levels of empowerment would be more likely to have a healthy sun-tanning behaviour, as in accordance with previous studies [19, 41]. Our study found that empowerment had a significant effect in the multivariate analysis (Table 5), and therefore contributes to the explanation of Norwegians’ sun-tanning behaviour. Furthermore, our results show that individuals with a high degree of perceived empowerment are more likely to engage in sun protective behaviour. This is in compliance with research showing that an empowered person is a person who take better care of themselves and their health [41, 42].

Several studies report that women more frequently sunbathe or have more high risk sun-tanning behaviour than men [9, 12, 31, 43, 44]. Our study found however that men scored higher regarding high risk sun-tanning behaviour. Men and women had the same score when it came to sun exposure. However, our study revealed that women found it more severe to be slightly sunburnt in order to get a sun tan, and they also reported more use of all three prevention strategies, i.e. sunscreen, clothing and seeking the shade. The latter is in agreement with previous findings [33, 45].

In the current study sun-tanning behaviour is designed in a way that individuals get a high sun-tanning behaviour score if they report high sun exposure, low sun protective behaviour and high tolerance of sunburn. Each question is “weighted” equally in the dependent variable, i.e., no factor is assumed more important in predicting sun-tanning behaviour. Despite the fact that sunscreen is recommended as the third best strategy for sun protective behaviour and a supplement to the other forms of protection [46], the use of sunscreen gave the same score for sun protection as wearing clothes or seeking the shade. The use of sunscreen as a means of reducing the negative health effects can be questioned. Previous research found that sunscreen users had more sunburn, more frequent sunbathing vacations and were more likely to use indoor tanning devices [4]. This trend is sometimes called the *sunscreen paradox* [10]. Sunscreen users often report prolonged sun exposure and thereby increased risk of sunburn and melanoma [46]. In general, this study concentrates on perceived tanning behaviour and does not contain detailed information about individuals’ use of protection or their actual sun exposure and therefore there is no rationale for weighting sunscreen use or any other component higher or lower than the other variables. It would be interesting to investigate further in future studies whether individuals who use sunscreen actually have healthier tanning behaviour.

### Strengths and limitations

A strength in this study is the large sample size, which makes it possible to draw general conclusions concerning the Norwegian population. A growing number of studies support the use of online research methods in order to promote health related behavioural change. We believe our study has covered the most important factors influencing the Norwegians’ sun-tanning behaviour. In order to minimize uncertainties and secure a deeper and more complete coverage of the area, the questionnaire was based on scaled answers. Another strength of this study is the inclusion of comprehensive preliminary tests that were conducted in order to ensure that there was no violation of the assumptions in the multiple regression. The assumptions are all concluded from the requirement that the model is correctly specified.

The study has some limitations. A response rate of 33% may imply bias regarding who has finished the questionnaire, i.e. these persons may be those with more “correct” and healthier behaviour. Retrospective survey items, which require recalling behaviour from the last 12 months and generalizing typical behaviour, may cause imprecisions and deviation in the measures [47]. Another limitation is that only selected aspects of beliefs, sun exposure and sun-tanning behaviour are covered in our questionnaire. Additional questions on the use of sunscreen and reapplication of it, could possibly determine whether or not individuals use sunscreen in order to prolong their time in the sun and thereby have a more unhealthy sun-tanning behaviour. A longer questionnaire however, can be too comprehensive and thereby causing bias by reduced response rate or inaccurate answers if the respondents rush through the survey in order to complete it.

### Implications for practice and research

Based on this study’s findings, it may be recommended for health communication and preventive work to target perceived barriers to sun protection as well as to increase knowledge and perception about the severity of melanoma. Changing people’s perceptions about the attractiveness of having tanned skin, could also be valuable in order to reduce the melanoma incidence. Previous research focusing on personalized risks and appearance related risks have shown promising results in terms of changing people’s intentions to sunbathing and using sun protection [48–50]. Furthermore, based on the significant effects empowerment showed in the analysis, our study suggests the possibility of incorporating empowerment strategies into future skin cancer interventions. Empowerment involves stronger involvement from the individual, and engaging people in their own self-management and behaviour change is critical to health service management and to governance more generally [51]. Successful implementation of empowering strategies can strengthen the whole individual, its autonomy, self-efficacy and general control, in achieving better and healthier lives [42], and thus a healthier sun-tanning behaviour.

## Conclusion

Norway is one of the countries with the highest incidence, mortality and healthy years lost due to melanoma, and sun-tanning behaviour has been suggested as the reason. To investigate the Norwegians sun-tanning behaviour and better understand perceptions, attitudes and behaviour in relation to sun-tanning, this study incorporated different attitudinal factors to the original Health Belief Model (HBM). The model was modified by adding the variables perceived empowerment and the benefits of tanning as separate factors. The comprehensive HBM explained 31% of the variance in sun-tanning behaviour, where perceived barriers to sun protection was shown to be the most important factor. This was followed by perceived severity and the benefits of tanning. Individuals’ perceived empowerment proved to have a significant impact on sun-tanning behaviour. Based on this study’s findings we suggest health promotion policies and preventive work to emphasize on reducing barriers to sun protection, increasing knowledge about healthy sun-tanning behaviours and the detrimental effects of tanning. Finally, using an empowerment approach may not only strengthen the individual’s decision-making in relation to protective sun behaviours, but also improve their overall health.

## Additional file

Additional file 1: Questionnaire including a list of variables used in data analysis. (PDF 201 kb)

## Abbreviations

HBM: Health Belief Model
UV: Ultraviolet radiation
