# Supplementary material for: Beliefs, attitudes and perceptions to sun-tanning behaviour in the Norwegian population: a cross-sectional study using the health belief model
Source: BMC Public Health. 2019 Feb 18;19:206. doi: 10.1186/s12889-019-6503-0 (PMC6380037; doi:10.1186/s12889-019-6503-0)
Supplement: Supplementary file 1 — Questionnaire including a list of variables used in data analysis. (PDF 201 kb) [file 12889_2019_6503_MOESM1_ESM.pdf]

**Questionnaire applied in Norwegian, Translated to English.**
**A list of variables is presented at the bottom of the document.**

# Questionnaire on sun behaviour

*This survey was developed into an electronic questionnaire by the market research company Norstat. The respondents received questions about participation by mail, and could click on the attached link to proceed to the questionnaire. The survey was set up in a well-organized web design where all questions had to be answered before one could move on to the next.*

**Information to the respondents on the first page of the questionnaire:**
This study focuses on intentional tanning, which means when you seek out the sun with the intention to sunbathe and to achieve a tan. For example, in sunbeds, on the beach and going on vacation to sunny areas.

## Background

1. **Gender**

   ☐ Female

   ☐ Male

2. **Birth year** (Example 1980)

   _________

3. **In which county do you live in?**
   _______ (drop-down menu with choices)

4. **What is your highest terminated education?**

   ☐ No education or less than 9 years primary school

   ☐ Primary education

   ☐ High school

   ☐ College degree or Vocational college?

   ☐ Higher education (up to 2 years)

   ☐ Higher education (4 years + Masters degree or equivalent)

   ☐ Higher education (6 years + PhD or equivalent)

   ☐ Other

5. **What is your current work situation? (check several if applicable)**

☐ Student

☐ At home

☐ Jobseeker / laid off

☐ Vocational rehabilitation/disabled

☐ Retired

☐ Employed in the public sector

☐ Employed in a private business

☐ Self-employed

☐ Family member without fixed salary within a family business (example: farm)

☐ Other

6. **What was your annual income (before tax) over the past year**?
(including. child support, unemployment insurance, cash support, etc.)

☐ Under 200.000 NOK

☐ 200 000 – 299 999 NOK

☐ 300 000 – 399 999 NOK

☐ 400 000 – 499 999 NOK

☐ 500 000 – 749 999 NOK

☐ 750 000 – 999 999 NOK

☐ 1 000 000 NOK and above

7. **What was your natural hair colour when you were 18 years old?**

☐ Black

☐ Dark brown

☐ Light brown

☐ Blond

☐ Red

8. **Look at your right arm, from the shoulder and down to the wrist, approximately how many moles do you have that is 2 mm in diameter or more?**

☐ None

☐ 1-5

☐ 6-10

☐ 11-15

☐ 16-20

☐ 21-25

☐ More than 25

**9. Have you or any of your biological family ever been diagnosed with skin cancer/ melanoma?**

☐ No, none in my family

☐ Yes, a family member

☐ Yes, several family members

☐ I do not know / uncertain

**10. Has anyone in your circle of acquaintances been diagnosed with melanoma? (example: friends, co-workers)**

☐ No, none in my circle of acquaintances

☐ Yes, one

☐ Yes, several

☐ I do not know / uncertain

**11. If you sunbathe in the beginning of the summer without using sun protection, how does your skin react?**

☐ Brown/tanned without first getting red

☐ Red

☐ Red with a burning sensation

☐ Red with sunburn and blistering

**12. How is your skin after repeated sunbathing over a long time?**

☐ I get a deep tan

☐ I get a tan

☐ I get slightly tanned

☐ I never get a tan

## Sun behaviour

**13. Do you seek the sun to sunbathe/get a tan when you are home in Norway and/or on vacation?**

☐ No, I do not seek the sun

☐ Yes, I seek the sun

NOTE! Respondents who answer "No, I do not seek sun" on question 13 will not receive question: 14, 15, 26, 27, 28, 30, 31, 32

**14. If you answered yes, how much do you protect yourself from the sun in the following ways when you sunbathe?**

| | **Never** (0% of the time) | **Seldom** (10-20% of the time) | **Sometimes** (30-50% of the time) | **Often** (60-70% of the time) | **Always** (90-100% of the time) |
|---|---|---|---|---|---|
| **Sunscreen** | ☐ | ☐ | ☐ | ☐ | ☐ |
| **Clothing** (covering parts of your body) | ☐ | ☐ | ☐ | ☐ | ☐ |
| **Take breaks** in the shadow or indoors | ☐ | ☐ | ☐ | ☐ | ☐ |

**15. Recall a week this summer when it was nice weather in Norway. How many days did you sunbathe for 30 minutes or more?**
**(We want to know approximately how frequent you sunbathed, also without clear sun the whole week)**

☐ None

☐ 1 day

☐ 2 days

☐ 3 days

☐ 4 days

☐ 5 days

☐ 6 days

☐ 7 days, everyday

**16. How many weeks have you been on "sunny holidays" during the past 12 months?** (with "sunny holiday" we mean traveling to countries such as Spain, Greece, Turkey, Italy, Thailand etc.)

☐ None

☐ Less than a week

☐ 1 week

☐ 2 weeks

☐ 3 weeks

☐ 4 weeks or more

**17. How many times did you use a sunbed during the past 12 months?**

☐ I have never used a sunbed

☐ None (but I have previously used a sunbed)

☐ About ____ number of times

# Perceived risk and consequences of sunbathing

Below are some statements about the risks and consequences of sunbathing. Please mark to which degree you disagree or agree with the following statements.

**18. How likely do you think it is that you will be sunburned next summer and/or on your next vacation?**

Very unlikely — Very likely

| 0 | 1 | 2 | 3 | 4 | 5 | 6 | 7 | 8 | 9 | 10 |
|---|---|---|---|---|---|---|---|---|---|---|
| ☐ | ☐ | ☐ | ☐ | ☐ | ☐ | ☐ | ☐ | ☐ | ☐ | ☐ |

**19. How likely do you think it is that you want to develop melanoma during your lifetime?**

Very unlikely — Very likely

| 0 | 1 | 2 | 3 | 4 | 5 | 6 | 7 | 8 | 9 | 10 |
|---|---|---|---|---|---|---|---|---|---|---|
| ☐ | ☐ | ☐ | ☐ | ☐ | ☐ | ☐ | ☐ | ☐ | ☐ | ☐ |

**20. Compared to others at your age, what is the likelihood of you developing melanoma during your lifetime (or get melanoma again, if you have already had one)?**

| Far below the average | | | | | Average | | | | | Far above the average |
|---|---|---|---|---|---|---|---|---|---|---|
| 0 | 1 | 2 | 3 | 4 | 5 | 6 | 7 | 8 | 9 | 10 |
| ☐ | ☐ | ☐ | ☐ | ☐ | ☐ | ☐ | ☐ | ☐ | ☐ | ☐ |

Below are some statements. Please mark to which degree you disagree or agree with the them:

**21. I believe getting a sunburn is serious**

| Strongly disagree | | | | | Neutral | | | | | Strongly agree |
|---|---|---|---|---|---|---|---|---|---|---|
| 0 | 1 | 2 | 3 | 4 | 5 | 6 | 7 | 8 | 9 | 10 |
| ☐ | ☐ | ☐ | ☐ | ☐ | ☐ | ☐ | ☐ | ☐ | ☐ | ☐ |

**22. Developing melanoma would have serious consequences for me**

| Strongly disagree | | | | | Neutral | | | | | Strongly agree |
|---|---|---|---|---|---|---|---|---|---|---|
| 0 | 1 | 2 | 3 | 4 | 5 | 6 | 7 | 8 | 9 | 10 |
| ☐ | ☐ | ☐ | ☐ | ☐ | ☐ | ☐ | ☐ | ☐ | ☐ | ☐ |

**23. It is important for me to prevent getting melanoma**

| Strongly disagree | | | | | Neutral | | | | | Strongly agree |
|---|---|---|---|---|---|---|---|---|---|---|
| 0 | 1 | 2 | 3 | 4 | 5 | 6 | 7 | 8 | 9 | 10 |
| ☐ | ☐ | ☐ | ☐ | ☐ | ☐ | ☐ | ☐ | ☐ | ☐ | ☐ |

**24. Developing melanoma in the future worries me**

| Strongly disagree | | | | | Neutral | | | | | Strongly agree |
|---|---|---|---|---|---|---|---|---|---|---|
| 0 | 1 | 2 | 3 | 4 | 5 | 6 | 7 | 8 | 9 | 10 |
| ☐ | ☐ | ☐ | ☐ | ☐ | ☐ | ☐ | ☐ | ☐ | ☐ | ☐ |

**25. I think it is easy to treat melanoma**

| Strongly disagree | | | | | Neutral | | | | | Strongly agree |
|---|---|---|---|---|---|---|---|---|---|---|
| 0 | 1 | 2 | 3 | 4 | 5 | 6 | 7 | 8 | 9 | 10 |
| ☐ | ☐ | ☐ | ☐ | ☐ | ☐ | ☐ | ☐ | ☐ | ☐ | ☐ |

## Reasons for using sun protection

Below are some statements of different types of sun protection. Please mark to which degree you disagree or agree with them:

**26. When I am going to sunbathe, using sun screen is...**

| | Strongly disagree | Disagree | Neutral | Agree | Strongly agree |
|---|---|---|---|---|---|
| Uncomfortable | ☐ | ☐ | ☐ | ☐ | ☐ |
| Inconvenient | ☐ | ☐ | ☐ | ☐ | ☐ |
| Too expensive | ☐ | ☐ | ☐ | ☐ | ☐ |
| Too time consuming | ☐ | ☐ | ☐ | ☐ | ☐ |
| To put on sunscreen is not something I like to do | ☐ | ☐ | ☐ | ☐ | ☐ |
| Something I often forget | ☐ | ☐ | ☐ | ☐ | ☐ |
| Something that prevents me from achieving the tan I want | ☐ | ☐ | ☐ | ☐ | ☐ |

**27. When I am going to sunbathe, using clothes to protect me from the sun is...**

| | Strongly disagree | Disagree | Neutral | Agree | Strongly agree |
|---|---|---|---|---|---|
| Uncomfortable | ☐ | ☐ | ☐ | ☐ | ☐ |
| Inconvenient | ☐ | ☐ | ☐ | ☐ | ☐ |

| | | | | | |
|---|---|---|---|---|---|
| Embarrassing | ☐ | ☐ | ☐ | ☐ | ☐ |
| Something I do not like | ☐ | ☐ | ☐ | ☐ | ☐ |
| Something that prevents me from achieving the tan I want | ☐ | ☐ | ☐ | ☐ | ☐ |

**28. When I am going to sunbathe, taking breaks in the shadows or indoors is…**

| | Strongly disagree | Disagree | Neutral | Agree | Strongly agree |
|---|---|---|---|---|---|
| Inconvenient | ☐ | ☐ | ☐ | ☐ | ☐ |
| Something that prevents me from achieving the tan I want | ☐ | ☐ | ☐ | ☐ | ☐ |

# Statements in relation to sun protection

**29.** Below are some statements of sun protection. Please mark to which degree you disagree or agree with the following statements:

| | Strongly disagree | Disagree | Neutral | Agree | Strongly agree |
|---|---|---|---|---|---|
| By using sunscreen with a sun protection factor (SPF) of 15 or more, one can avoid being sunburned | ☐ | ☐ | ☐ | ☐ | ☐ |
| By staying in the shade, the chance of getting a sunburn is reduced | ☐ | ☐ | ☐ | ☐ | ☐ |
| By avoiding the sun between 12-15 pm, one can avoid being sunburned | ☐ | ☐ | ☐ | ☐ | ☐ |
| By using sunscreen with SPF 15 or more while in the sun, the chances of getting melanoma is reduced | ☐ | ☐ | ☐ | ☐ | ☐ |
| By staying in the shade, the chance of getting melanoma is reduced | ☐ | ☐ | ☐ | ☐ | ☐ |

| | | | | | |
|---|---|---|---|---|---|
| By using clothes when in the sun, the chance of getting melanoma is reduced | ◻ | ◻ | ◻ | ◻ | ◻ |

## Opinions about sunbathing

*For those who answered "No, I do not seek the sun" on question 13:*

Please mark to which degree do you disagree or agree on the following statements:

**30. I avoid sunbathing because...**

| | Strongly disagree | Disagree | Neutral | Agree | Strongly agree |
|---|---|---|---|---|---|
| I'm afraid of getting skin cancer/ melanoma | ◻ | ◻ | ◻ | ◻ | ◻ |
| I do not want to get wrinkles or pigment changes/spots on my skin | ◻ | ◻ | ◻ | ◻ | ◻ |
| I don't have time or don't want to spend time sunbathing | ◻ | ◻ | ◻ | ◻ | ◻ |
| It's boring, uncomfortable, too hot/ sweaty | ◻ | ◻ | ◻ | ◻ | ◻ |
| None of my friends/family sunbathe | ◻ | ◻ | ◻ | ◻ | ◻ |
| I do not want to get a tan | ◻ | ◻ | ◻ | ◻ | ◻ |
| I do not want to get sunburned | ◻ | ◻ | ◻ | ◻ | ◻ |

*For those who answered "Yes, I seek the sun" on question 13:*

Below are some reasons why people want to tan/sunbathe. Please mark to which degree do you disagree or agree on the following statements:

**31. I sunbathe because…**

| | Strongly disagree | Disagree | Neutral | Agree | Strongly agree |
|---|---|---|---|---|---|
| I find it comfortable | ◻ | ◻ | ◻ | ◻ | ◻ |

| | | | | | |
|---|---|---|---|---|---|
| I feel better with a tanned skin | ☐ | ☐ | ☐ | ☐ | ☐ |
| I look better when I am tanned | ☐ | ☐ | ☐ | ☐ | ☐ |
| It is social to sunbathe with friends / family | ☐ | ☐ | ☐ | ☐ | ☐ |
| My friends sunbathe and have a tanned skin | ☐ | ☐ | ☐ | ☐ | ☐ |
| I want supply of vitamin D | ☐ | ☐ | ☐ | ☐ | ☐ |
| I need a tan for my job, sport activity or hobby | ☐ | ☐ | ☐ | ☐ | ☐ |

**32. It is worth getting slightly sunburned to get a tan**

Strongly disagree — Neutral — Strongly agree

| 0 | 1 | 2 | 3 | 4 | 5 | 6 | 7 | 8 | 9 | 10 |
|---|---|---|---|---|---|---|---|---|---|---|
| ☐ | ☐ | ☐ | ☐ | ☐ | ☐ | ☐ | ☐ | ☐ | ☐ | ☐ |

Below are some reasons for why people limit their sunbathing/sun exposure. Please mark to which degree you agree or disagree with the following statements:

**33. I limit my sunbathing because...**

| | Strongly disagree | Disagree | Neutral | Agree | Strongly agree |
|---|---|---|---|---|---|
| I'm afraid of getting skin cancer | ☐ | ☐ | ☐ | ☐ | ☐ |
| I do not want to get wrinkles or pigmentations in the skin | ☐ | ☐ | ☐ | ☐ | ☐ |
| I don't have time or don't want to spend time sunbathing | ☐ | ☐ | ☐ | ☐ | ☐ |
| It's boring, uncomfortable, too hot/ sweaty | ☐ | ☐ | ☐ | ☐ | ☐ |
| None of my friends or family sunbathe | ☐ | ☐ | ☐ | ☐ | ☐ |
| I do not want to get sunburned | ☐ | ☐ | ☐ | ☐ | ☐ |

## Perceived control and influence over own life

**34. Overall, how much control do you feel you have, about whether you get skin cancer in the future?**

| No control | | | | | | | | | | Full control |
|---|---|---|---|---|---|---|---|---|---|---|
| 0 | 1 | 2 | 3 | 4 | 5 | 6 | 7 | 8 | 9 | 10 |
| ☐ | ☐ | ☐ | ☐ | ☐ | ☐ | ☐ | ☐ | ☐ | ☐ | ☐ |

**35. I am able to detect warning signs of melanoma at an early stage**

| Not capable of detecting warning signs | Not sure | | | | | | | | | Absolutely sure |
|---|---|---|---|---|---|---|---|---|---|---|
| 0 | 1 | 2 | 3 | 4 | 5 | 6 | 7 | 8 | 9 | 10 |
| ☐ | ☐ | ☐ | ☐ | ☐ | ☐ | ☐ | ☐ | ☐ | ☐ | ☐ |

**36. I feel confident about what to look for when checking my skin for warning signs of melanoma**

| Do not know what to look for | Not sure | | | | | | | | | Absolutely sure |
|---|---|---|---|---|---|---|---|---|---|---|
| 0 | 1 | 2 | 3 | 4 | 5 | 6 | 7 | 8 | 9 | 10 |
| ☐ | ☐ | ☐ | ☐ | ☐ | ☐ | ☐ | ☐ | ☐ | ☐ | ☐ |

**37. I feel I know what to do to avoid getting sunburned when I sunbathe**

| Do not know what to do to avoid a sunburn | Not sure | | | | | | | | | Absolutely sure |
|---|---|---|---|---|---|---|---|---|---|---|
| 0 | 1 | 2 | 3 | 4 | 5 | 6 | 7 | 8 | 9 | 10 |
| ☐ | ☐ | ☐ | ☐ | ☐ | ☐ | ☐ | ☐ | ☐ | ☐ | ☐ |

**38. I am certain that I will protect myself against the sun, even though those accompanying me do not**

| Not sure | | | | | | | | | Absolutely sure |
|---|---|---|---|---|---|---|---|---|---|
| 1 | 2 | 3 | 4 | 5 | 6 | 7 | 8 | 9 | 10 |
| ☐ | ☐ | ☐ | ☐ | ☐ | ☐ | ☐ | ☐ | ☐ | ☐ |

Please mark to which degree you disagree or agree with the following statements:

**39. I aim to protect myself against the sun to avoid sunburn, also when it may be impracticable or difficult**

Strongly disagree Strongly agree

| 0 | 1 | 2 | 3 | 4 | 5 | 6 | 7 | 8 | 9 | 10 |
|---|---|---|---|---|---|---|---|---|---|---|
| ☐ | ☐ | ☐ | ☐ | ☐ | ☐ | ☐ | ☐ | ☐ | ☐ | ☐ |

**40. I feel that I have the freedom to make my own choices and have influence over my own life**

Strongly disagree Strongly agree

| 0 | 1 | 2 | 3 | 4 | 5 | 6 | 7 | 8 | 9 | 10 |
|---|---|---|---|---|---|---|---|---|---|---|
| ☐ | ☐ | ☐ | ☐ | ☐ | ☐ | ☐ | ☐ | ☐ | ☐ | ☐ |

**41. I have a positive attitude towards myself**

Strongly disagree Strongly agree

| 0 | 1 | 2 | 3 | 4 | 5 | 6 | 7 | 8 | 9 | 10 |
|---|---|---|---|---|---|---|---|---|---|---|
| ☐ | ☐ | ☐ | ☐ | ☐ | ☐ | ☐ | ☐ | ☐ | ☐ | ☐ |

**42. I usually trust the decisions I make**

Strongly disagree Strongly agree

| 0 | 1 | 2 | 3 | 4 | 5 | 6 | 7 | 8 | 9 | 10 |
|---|---|---|---|---|---|---|---|---|---|---|
| ☐ | ☐ | ☐ | ☐ | ☐ | ☐ | ☐ | ☐ | ☐ | ☐ | ☐ |

**43. I often feel powerless**

Strongly disagree Strongly agree

| 0 | 1 | 2 | 3 | 4 | 5 | 6 | 7 | 8 | 9 | 10 |
|---|---|---|---|---|---|---|---|---|---|---|
| ☐ | ☐ | ☐ | ☐ | ☐ | ☐ | ☐ | ☐ | ☐ | ☐ | ☐ |

**44. I am a person who takes responsibility/action of a problem when it occurs**

| Strongly disagree | | | | | | | | | | Strongly agree |
| --- | --- | --- | --- | --- | --- | --- | --- | --- | --- | --- |
| 0 | 1 | 2 | 3 | 4 | 5 | 6 | 7 | 8 | 9 | 10 |
| ☐ | ☐ | ☐ | ☐ | ☐ | ☐ | ☐ | ☐ | ☐ | ☐ | ☐ |

**Thank you very much for your participation!**

If you want to learn about sun protection and melanoma, you can visit the Norwegian Cancer Society's and Helsenorge's websites.

The Norwegian Cancer Society:
https://kreftforeningen.no/forebygging/sol-solarium-og-kreft

Helsenorge:
https://helsenorge.no/sykdom/kreft/hudkreft
https://helsenorge.no/sykdom/hud-og-har/soling-solkrem-og-uv-straler

List of variables used in “Beliefs, attitudes and perceptions to sun-tanning behaviour in the Norwegian population. A cross-sectional study using the Health Belief Model”

| | |
|---|---|
| **Outcome variable** | |
| Sun-tanning behaviour | **Question**<br>- 14 (3 items, all of them with reversed direction, i.e., “Always” assigned to “low score” and “Never” assigned to “high score”)<br>- 15<br>- 16<br>- 17<br>- 32 |
| **Predictor variables** | |
| **Susceptibility** | Question<br>- 18<br>- 19<br>- 20 |
| **Severity** | Question<br>- 21<br>- 22<br>- 23<br>- 24<br>- 25 (with reversed direction from “Strongly agree” assigned to “low score” and “Strongly disagree” assigned to “high score”) |
| **Benefits (of using sun protection)** | Question<br>- 29 (6 items) |
| **Barriers (of using sun protection)** | Question<br>- 26 (7 items)<br>- 27 (5 items)<br>- 28 (2 items) |
| **Self-efficacy** | Question<br>- 34<br>- 35<br>- 36<br>- 37<br>- 38<br>- 39 |

| | |
|---|---|
| **Empowerment** | Question<br>- 40<br>- 41<br>- 42<br>- 43 (with reversed direction from “Strongly agree” assigned to “low score” and “Strongly disagree” assigned to “high score”)<br>- 44 |
| **Benefits of tanning** | Question<br>- 31 (7 items) |
